# 20 Years of Pediatric Benchmarking in Germany and Austria: Age-Dependent Analysis of Longitudinal Follow-Up in 63,967 Children and Adolescents with Type 1 Diabetes

**DOI:** 10.1371/journal.pone.0160971

**Published:** 2016-08-17

**Authors:** Barbara Bohn, Beate Karges, Christian Vogel, Klaus-Peter Otto, Wolfgang Marg, Sabine E. Hofer, Elke Fröhlich-Reiterer, Martin Holder, Michaela Plamper, Martin Wabitsch, Wolfgang Kerner, Reinhard W. Holl, DPV Initiative

**Affiliations:** 1 Institute of Epidemiology and Medical Biometry, ZIBMT, University of Ulm, Ulm, Germany; 2 German Center for Diabetes Research (DZD), Munich-Neuherberg, Germany; 3 Division of Endocrinology and Diabetes, Medical Faculty, RWTH Aachen University, German Center for Diabetes Research (DZD), Aachen, Germany; 4 Department of Gynecological Endocrinology and Reproductive Medicine, Medical Faculty, RWTH Aachen University, German Center for Diabetes Research (DZD), Aachen, Germany; 5 Department of Pediatrics, Bethlehem Hospital, Stolberg, Germany; 6 Department of Pediatrics, Endocrinology and Diabetology, Clinic Chemnitz, Chemnitz, Germany; 7 Center for Pediatrics and Adolescents Medicine, Neonatology and Pediatric intensive care, Clinic Itzehoe, Itzehoe, Germany; 8 Center for Pediatrics and Adolescent Medicine, Bremen-Mitte Hospital, Bremen, Germany; 9 Department of Paediatrics, Medical University of Innsbruck, Innsbruck, Austria; 10 Department of Pediatrics, Medical University of Graz, Graz, Austria; 11 Department of Pediatric Endocrinology and Diabetology, Olgahospital, Stuttgart Clinical Center, Stuttgart, Germany; 12 Department of Pediatric Endocrinology and Diabetology, University Hospital Bonn, Bonn, Germany; 13 Division of Pediatric Endocrinology and Diabetes, University Hospital for Children and Adolescents, Ulm, Germany; 14 Centre of Diabetes and Metabolic Disorders, Karlsburg, Germany; University of Colorado Denver School of Medicine, UNITED STATES

## Abstract

**Background:**

To investigate changes in diabetes treatment over the last two decades in three age-groups of children and adolescents with type 1 diabetes (T1D) from Germany and Austria.

**Methods:**

63,967 subjects (<18yr) with T1D documented between 1995 and 2014 from the DPV-database were included and stratified according to age (0.5-<6, 6-<12, 12-<18yr). Regression models were applied for insulin regimens (<3 and ≥4 injection time points/day, or continuous subcutaneous insulin infusion (CSII)), use of rapid- and long acting insulin analogues, NPH insulin, and frequency of self-monitoring of blood glucose (SMBG)/day. Models were adjusted for sex, diabetes duration, and migration background. P-value for trend was given.

**Findings:**

The number of subjects with <3 injection time points/day decreased from 1995 to 2014 to <5% in all age-groups (p<0.0001). Proportion of patients with ≥4 injections/day increased until the early 2000s, and then declined until 2014. This trend was not found in 6-<12yr olds (p = 0.3403). CSII increased in all age-groups (p<0.0001) with the highest increase in children <6 years (from 0.4% to 79.2%), and the lowest increase in 12-<18 year olds (from 1.0% to 38.9%). NPH insulin decreased in all age-groups (p<0.0001). Insulin analogues, especially rapid-acting, became more frequent in all age-groups (p<0.0001), accounting for 78.4% in 2014 for all subjects. The highest use was found in the youngest children (in 2014: 85.6%), the lowest use in 6-<12 year olds (in 2014: 72.9%). The number of SMBG/day increased from 2.2 to 6.4 with a similar rise in all age-groups (p<0.0001). Frequency was highest in subjects <6yr.

**Conclusions:**

In all age-groups, T1D treatment was intensified over the last 20 years. Age-specific differences in trends were particularly observed in the number of patients on CSII, in the number of patients with 4 or more injections/day, and in the frequency of SMBG/day.

## Introduction

The German/Austrian Diabetes-Patienten-Verlaufsdokumentation (DPV) initiative was the first nationwide benchmarking launched in Germany in the year 1995. DPV focused initially on children and adolescents with diabetes and was extended to adult patients in 1997 [1]. The DPV initiative is based on three modules for diabetes documentation and quality management: 1. the DPV software which is used for continuous, longitudinal, prospective documentation of diabetes-related parameters, 2. external benchmarking for participating centres, and 3. a database for epidemiologic and medical research on diabetes (www.d-p-v.eu). Until today, DPV has been used for multiple aspects of patient-centered research including health care and economic analyses [2–4].

New technologies and the availability of insulin analogues led to major changes in the management of diabetes in children and adolescents [5]. Overall, an increase in intensified insulin therapy is reported in children and adolescents with type 1 diabetes [6–8]. However, time trends in diabetes therapy might differ between age-groups due to different needs and preferences. Medical conditions such as a high risk of hypoglycemia in very young children [9], worse metabolic control or a higher frequency of mental disorders in adolescents [10–12] may lead to different treatment regimens. Furthermore special challenges due to health care transition from pediatric to an adult diabetes care provider [13] and non-medical conditions have to be considered in the choice of diabetes therapy. There are large differences between parent-child and parent-adolescent relationships. The transfer from adolescents to adulthood is a phase of substantial changes including less time with their family, separation from parents and increasing independency [14,15]. Moreover, there are age-specific guideline recommendations for certain areas in the management of diabetes (e.g. pump use) [16,17].

Previous studies investigating trends in diabetes treatment in children and adolescent with type 1 diabetes are limited by a short study period or a low number of documented cases [7], a small country size [6,18], and recent studies were not population-based [10] or not stratified by age [8].

Therefore, we aimed to investigate changes in insulin treatment over the last two decades in three age-groups of children and adolescents with type 1 diabetes from Germany and Austria.

## Patients and Methods

### Ethics statement

Analysis of anonymized routine data within the German/Austrian DPV Initiative was approved by the Ethics Committee of the Medical Faculty of the University of Ulm, Germany.

### Data source and subjects

Data were provided by the DPV database which is currently used by 426 centers from Germany and Austria. Twice a year, anonymized data are transmitted from participating health care facilities to the study center Ulm, Germany and aggregated into a cumulative database for clinical research and quality assurance. Inconsistent data are reported back to the participating centers for confirmation or correction and are reentered into the database.

**Fig 1:**
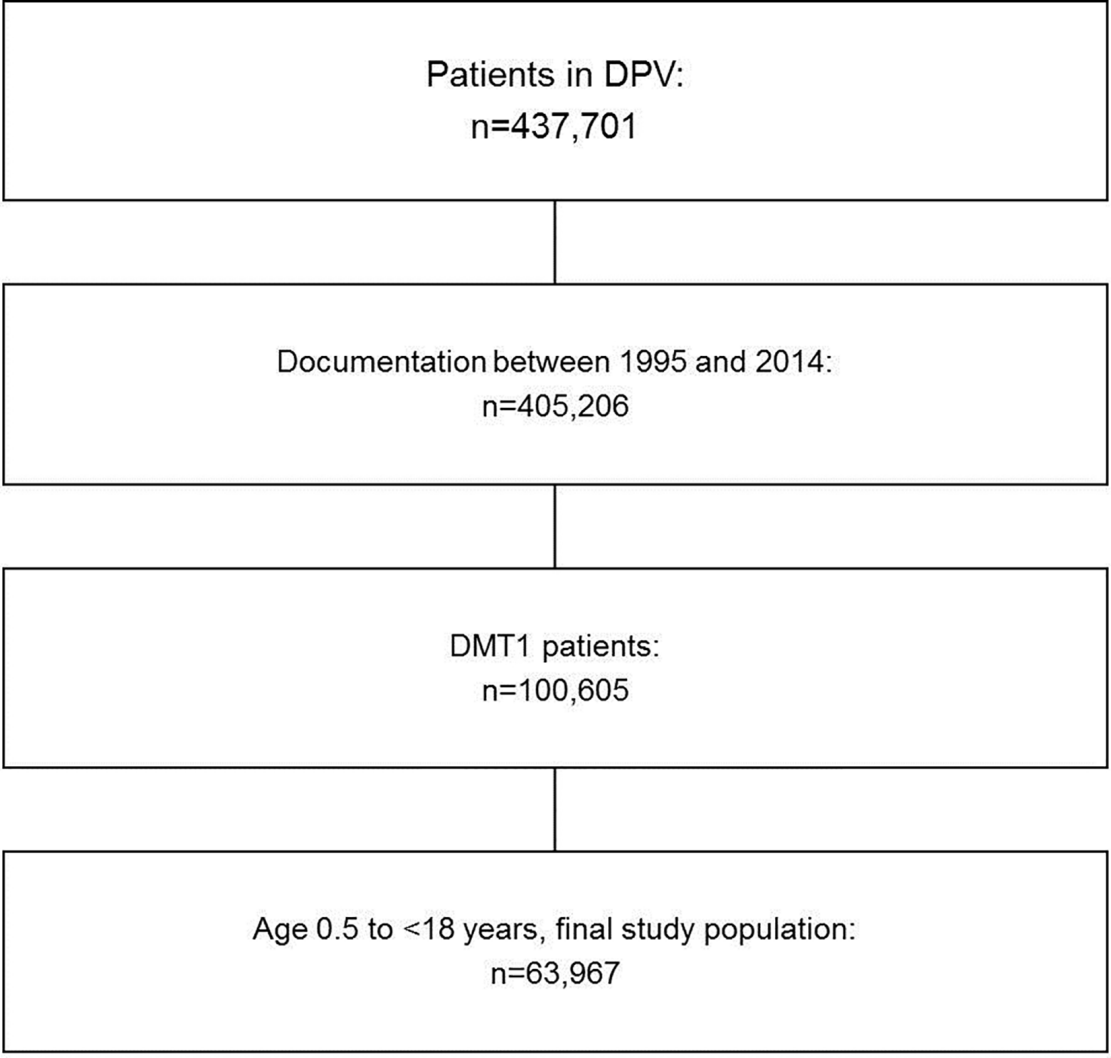
Selection of study population.

As of September 2015, 437,701 patients were registered in DPV. Patients with type 1 diabetes and an age between 0.5 and <18 years were included in the analysis (Fig 1). The study population was grouped according to age (0.5 to <6; 6 to <12, and 12 to <18 years). Due to the longitudinal character of this analysis, most patients are included in more than one calendar year (mean documentation period: 4.8 years).

### Outcome variables

Sociodemographic and clinical data included sex, age, age at diabetes diagnosis, diabetes duration, HbA_1C_, and body mass index (BMI). HbA_1C_ was mathematically standardized to the reference range of 20–42 mmol/mol (Diabetes Control and Complication Trial: 4.05–6.05%) by applying the multiple-of-the-mean transformation method [19]. BMI, expressed as weight in kilograms/squared height in meter (kg/m^2^) was given as standard deviation score (SDS), using reference data from a nationally representative sample of German adolescents [20].

We analyzed the self-reported frequency of insulin injections per day (1–2, 3, or ≥ 4 injection time points), use of insulin pumps, use of rapid- and long-acting insulin analogues, NPH (Neutral Protamine Hagedorn) insulin, and the frequency of self-monitoring of blood glucose (SMBG) per day. SMBG per day was also analyzed stratified by intensified conventional therapy (ICT, defined as 4 or more injection time points per day) or pump use.

### Statistical analysis

Descriptive statistics were implemented for the whole study population (Table 1). Sociodemographic and clinical characteristics were presented as median (Q1;Q3) or as percentage (%). In descriptive statistics, the most recent year of treatment was used for each patient.

**Table 1:** Sociodemographic and clinical characteristics of all subjects included and stratified according to age-groups.

	whole study population n = 63,967	<6 years of age n = 3,172	6-<12 years of age n = 13,601	12-<18 years of age n = 47,194
male, %	52.7	53.1	51.2	53.1
age [year]	15.4 (11.7;17.4)	4.4 (3.3;5.2)	9.7 (8.1;10.9)	16.7 (14.8;17.6)
age at diabetes diagnosis [year]	8.7 (5.1;11.9)	2.8 (1.8;3.9)	6.1 (3.8;8.2)	10.1 (6.6;12.8)
diabetes duration [year]	4.9 (2.1;8.2)	1.0 (0.2;2.1)	2.9 (1.1;5.3)	5.9 (3.0;9.4)
HbA_1C_ [%]	7.9 (7.0;9.1)	7.5 (6.8;8.3)	7.5 (6.8;8.3)	8.1 (7.2;9.4)
HbA_1c_ [mmol/mol]	62.8 (53.0;76.0)	58.5 (50.8;67.2)	58.5 (50.8;67.2)	65.0 (55.2;79.2)
BMI SDS	0.3 (-0.3;0.9)	0.5 (-0.2;1.1)	0.2 (-0.4;0.8)	0.3 (-0.3;0.9)

Data are unadjusted medians (Q1;Q3) unless otherwise indicated.

Logistic regression models were applied for dichotomous variables (1–2, 3, and ≥4 injections per day, use of insulin pumps, use of insulin analogues, and NPH insulin), linear regression for SMBG per day. Additionally, a generalized logistic model for insulin regimen (1–2, 3, 4 or more injection time points, and use of insulin pumps) as ordinal dependent variable was implemented. Results of regression analysis were given for the years 1995 to 2014, and for age-groups (0.5 to <6, 6 to <12, and 12 to <18 years), separately. All models were adjusted for sex, diabetes duration (categories: <2 years / ≥2 years), and migration background (children with at least one parent born outside of Germany). Regression models for the whole study population also included age as a confounding variable. P value for trend was calculated with calendar year as continuous variable.

Due to the large sample size, a two-sided p-value <0.01 was considered significant. All statistical analyses were implemented with SAS 9.4 (Statistical Analysis Software, SAS Institute, Cary, NC, USA).

## Results

**Fig 2:**
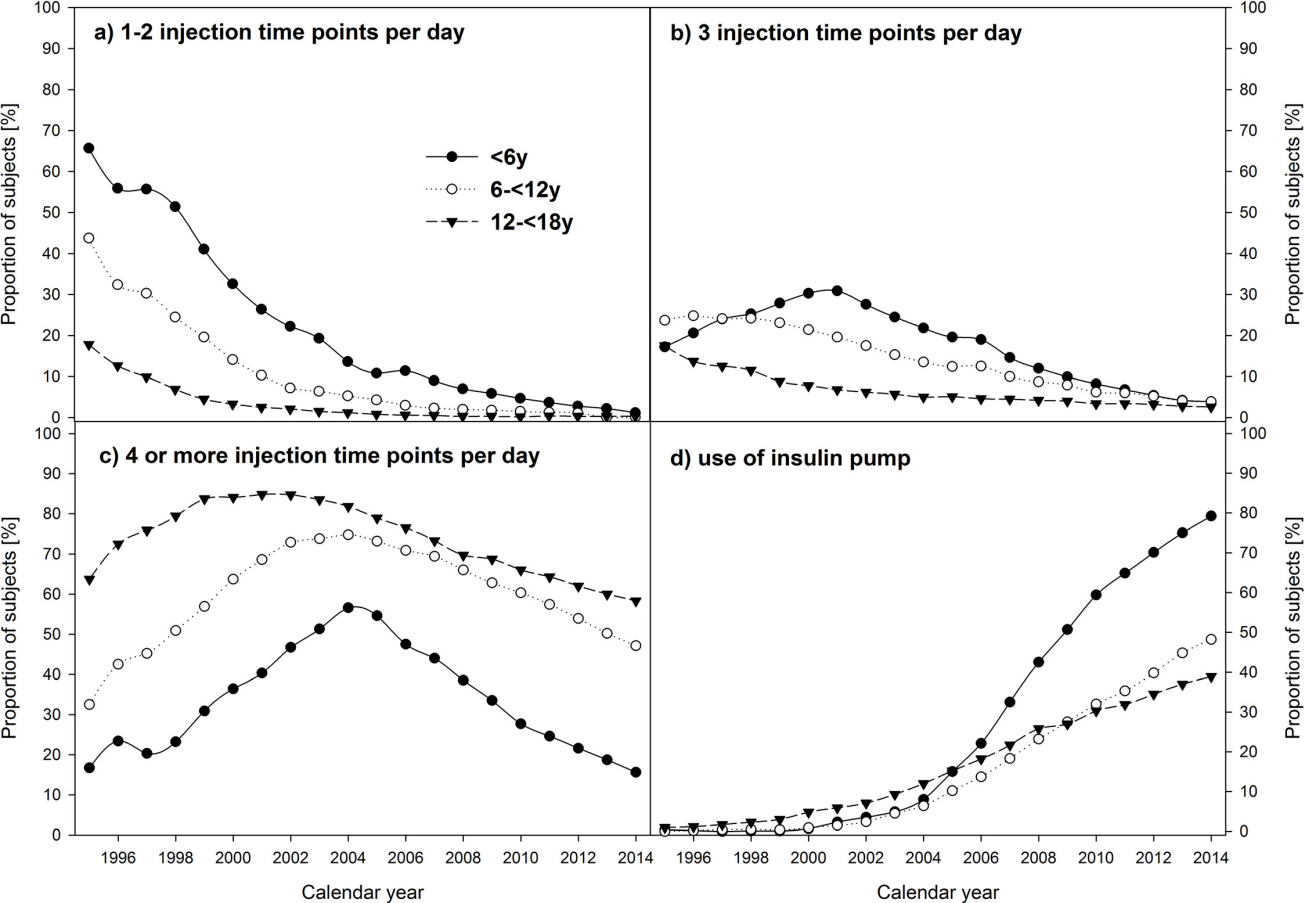
Frequency of a) 1–2 injections per day, b) 3 injections per day, c) 4 or more injections per day, and d) use of insulin pumps in pediatric patients with type 1 diabetes, stratified by calendar year and age-groups. Data adjusted for sex, diabetes duration, and migratory background.

**Fig 3:**
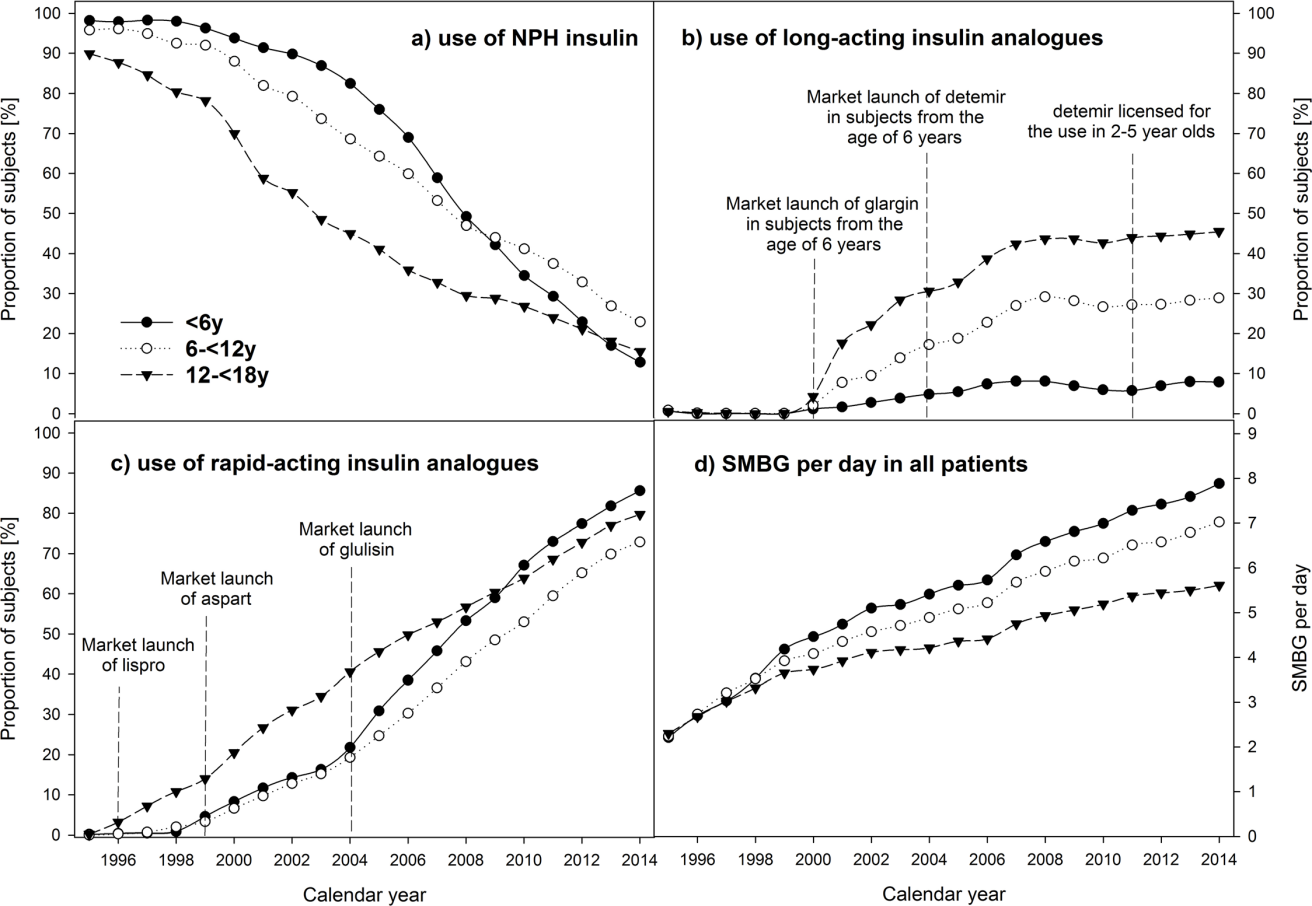
Use of a) NPH insulin, b) long-acting insulin analogues, c) rapid-acting insulin analogues, and d) SMBG per day in pediatric patients with type 1 diabetes, stratified by calendar year and age-groups. Data adjusted for sex, diabetes duration, and migratory background.

**Fig 4:**
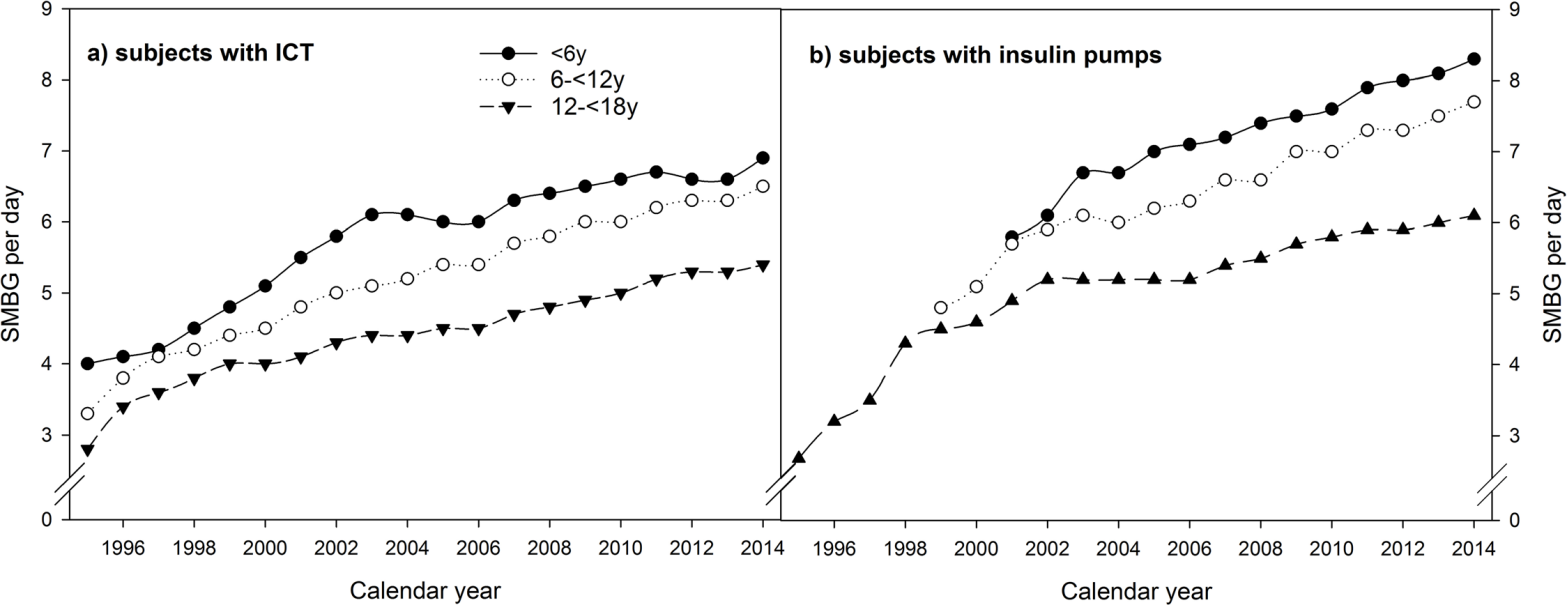
Frequency of SMBG per day in a) subjects with ICT, and b) subjects with insulin pumps, stratified by calendar year and age-groups. Data adjusted for sex, diabetes duration, and migratory background (data for small number of cases <20 are not shown).

We included a total of 63,967 children and adolescents with type 1 diabetes. Sociodemographic and clinical data are shown in Table 1. Trend analyses over the last two decades comprised a total of 305,844 patient-years and are illustrated in Figs 2–4.

### Trends in insulin regimens

Considering the whole study population, logistic regression analysis revealed a decrease in the number of patients with 1–2 injection time points per day (from 28.8% in 1995 to 0.4% in 2014) and 3 injection time points per day (from 17.8% to 2.8%) (both p<0.0001). This trend was present for all age-groups (Fig 2A and 2B, all p<0.0001). The number of patients with 4 or more injections per day initially increased from 52.8% in 1995 to 79.7% in 2002 and then declined again to 50.5% in 2014 (p<0.0001). This pattern was similar in children <6 years and in adolescents between 12 and 18 years of age (both p<0.0001), but not in 6 to 12 year olds (p = 0.3403) (Fig 2C). Over the last 20 years, the proportion of subjects using insulin pumps increased from 0.6% to 46.2% (p<0.0001). A large increase was observed in all age-groups, especially in the youngest (<6 years of age) accounting for 79.2% in 2014 (Fig 2D; all p<0.0001). These results were confirmed by the generalized logistic regression model with 4 insulin regimens of increasing intensity (p<0.0001).

### Trends in insulins and SMBG

The use of NPH insulin decreased in the whole study population from 93.4% in 1995 to 19.5% in 2014 and in all age-groups (Fig 3A, all p<0.0001). The use of rapid-acting insulin-analogues increased from 0.1% in 1995 to 78.4% in 2014, and long-acting insulin analogues from 0.7% to 34.3% (both p<0.0001). This trend was present in all age-groups (Fig 3B and 3C; all p<0.0001). In 2014, the highest use of long-acting insulin analogues (46%) was observed in adolescent patients. The number of SMBG per day rose from 2.2 in 1995 to 6.4 in 2014 in the whole study population with a similar rise in all age-groups (Fig 3D, all p<0.0001). The highest SMBG frequency was observed in subjects <6 years. In children and adolescents with ICT, SMBG per day increased from 3.2 in 1995 to 6.0 in 2014 and in subjects with insulin pumps from 3.0 to 6.9 per day (both p<0.0001). This trend was found in all age-groups (Fig 4A and 4B; all p<0.0001).

## Discussion

Our aim was to analyze trends of diabetes treatment in pediatric patients with type 1 diabetes in three age-groups (0.5 to <6, 6 to < 12, and 12 to <18 years) from Germany and Austria over the last two decades. In all age-groups, diabetes treatment was intensified and the type of insulins used changed substantially.

According to recommendations of national and international guidelines, a similar trend towards an intensified insulin treatment was found in all age-groups [16,21–23]. The proportion of subjects with <3 injection time points per day decreased to <5% until the year 2014 (Fig 2A and 2B). The overall decrease is consistent with findings from other studies [6–8,24,25]. However, these studies did not consider differences between age-groups; therefore no further comparison with our data is possible. Until the year 2004, the number of patients with 4 or more injection time points per day increased. With the increment of insulin pump use starting in the early 2000s, the proportion decreased again (Fig 2C and 2D). 4 or more injection time points per day were most frequent in the oldest age-group (58.3% in 2014), followed by children between 6 and 12 years (47.1% in 2014). By far, the lowest proportion was found in patients <6 years of age (15.6% in 2014). Accordingly, the strongest increase in the use of insulin pumps was found in this age group (from 0.4% in 1995 to 79.2% in 2014). In contrast, the lowest increase was present in the oldest patients (from 1.0% to 38.9%). Several factors could have contributed to these differences. German and some international guidelines of diabetes care in children and adolescents recommend the use of insulin pumps especially in infants and neonates [16,17,26]. Furthermore, emotional barriers (feeling uncomfortable or less attractive using an insulin pump) could be more relevant for adolescents compared to younger children. Additionally, age-dependent reimbursement decisions of health insurances might have contributed to the differences. An analysis of the most current registry data (2013/2014) of the American T1D exchange clinic registry also indicated a more frequent use of insulin pumps in 2-<6 (69%) and 6–12 year olds (68%) compared to 13–17 year olds (61%) [10]. The overall pump use in T1DX was higher compared to the frequency in the DPV study population. However, the authors stated that uninsured patients might be underreported in the T1D registry and therefore, pump use could be overestimated compared to the general population of children and adolescents with type 1 diabetes in the USA [10]. There are further studies investigating time trends in pump use, but without age-group analysis. In Sweden, an increase from 8% in 2001 to 37% in 2005 was reported [7]. In our study population, the proportion of subjects with insulin pumps was 13.5% in 2005 and increased to 46.2% in 2014. The lower percentage in the early 2000s is consistent with findings from a French study. In 2007pump use was reported in 12.7% [8].

In German guidelines, the use of regular human insulin or rapid-acting analogues is recommended [16]. Austrian recommendations are consistent with German guidelines. Our analysis indicated a clear preference in all age-groups towards the use of rapid-acting insulin analogues (Fig 3C). Moreover, our results revealed a slightly more frequent use in the youngest age-group. This can be explained by the higher proportion using insulin pumps in this age-group, because rapid-acting insulin analogues are recommended in pump use [16,17]. Overall, reasons for the superiority of rapid-acting insulin analogues might be more flexibility in the injection-meal-interval, or good experiences of physicians/patients [27]. Svensson and colleagues also reported a strong increase in the use of rapid-acting insulin analogues from 4% in 2000 to 58% in 2006 in Denmark [6]. Age-group analysis indicated the highest use in 10–18 year olds compared to 0–10 year olds (61% vs. 31%) [6]. Although age-groups differ from our study population, results are consistent to some extent. In 2006, the highest use (49.8%) was present in the oldest age-group (12-<18 years), followed by subjects below 6 years (38.5%). The lowest percentage was found in 6-<12 year olds (30.3%). DPV data for 2014 indicated the highest use in the youngest subjects.

Both, the use of NPH insulin or long-acting insulin analogues are recommended for basal insulin therapy [16]. In our study population, a strong decrease of NPH use was observed in all age-groups (from 90–98% in 1995 to 12–22% in 2014) (Fig 3A). For long-acting insulin analogues, trends differ between age-groups (Fig 3B). The highest increase was documented in the oldest, the lowest in the youngest age-group. The low percentage of children <6 years using long-acting insulin analogues is not surprising due to the high proportion on insulin pumps (79.2% in 2014). The stronger increase in the oldest age-group might also be explained by a higher risk of the Dawn phenomenon in adolescents. Moreover, differences in the licensing of long-acting insulin analogues need to be considered. In the year 2011, the European Medicines Agency expanded the license for the use of insulin detemir in 2–5 year olds. The overall increase is consistent with findings from other studies. A study from Sweden indicated an increment from 2% in 2004 to 23% in 2006 [6]. Since results were only given for the whole study population, further comparisons with our data are not possible.

According to the more intensive insulin treatment, the frequency of SMBG over the last two decades increased in all age-groups. This is consistent with other studies [6,28]. The large increase of SMBG per day is not only due to the more frequent use of insulin pumps. Stratification of the subjects according to ICT or insulin pump indicated a significant increment over the last 20 years in all groups (Fig 4A and 4B). Overall, the lowest frequency of SMBG per day was found in the oldest age-group, the highest in the youngest. This might be explained by more concerns of parents of toddlers or a greater negligence in adolescents [29].

### Strengths and limitations

This population-based analysis especially benefits from its large number of children and adolescents with type 1 diabetes from Germany and Austria. To our best knowledge, this is the first study investigating changes over two decades in pediatric diabetes treatment in three different age-groups. Analysis as the present one are urgently needed to assess age-specific differences in treatment changes. Another strength is the application of the same documentation software since 1995. Furthermore, detailed information on patients’ characteristics is available allowing to control for potential confounders.

One limitation is that the number of patients in the early years of the DPV registry is rather low. But in the course of the years, the number of participating DPV centers strongly increased. Now up to 90% of all pediatric patients with type 1 diabetes from Germany and more than 70% from Austria are registered in DPV. However, since this analysis does not constitute a full survey, a certain bias cannot be completely excluded. Moreover, independently of the amount of insulin analogues used within premixed insulin therapy, patients were assigned to the group “insulin analogues”. It should be also critically considered that the few patients combining nocturnal pump use with daily injections were classified as pump users. Another shortcoming might be that information on the frequency of SMBG per day is only in part downloaded and in part self-reported by patients.

## Conclusions

Substantial changes in the treatment of children and adolescents with type 1 diabetes from Germany and Austria over the last two decades are observed in all age-groups. Overall, insulin treatment was intensified, the use of NPH insulin strongly decreased, whereas the use of insulin analogues increased. Age-specific differences in trends were particularly present in the number of patients using insulin pumps, in the number of patients with 4 or more injection time points per day, in the use of long-acting insulin analogues as well as in the frequency of SMBG per day.

## Supporting Information

S1 Appendix Collaborating DPV centers. (PDF)
