# Supplementary material for: 20 Years of Pediatric Benchmarking in Germany and Austria: Age-Dependent Analysis of Longitudinal Follow-Up in 63,967 Children and Adolescents with Type 1 Diabetes
Source: PLoS One. 2016 Aug 17;11(8):e0160971. doi: 10.1371/journal.pone.0160971 (PMC4988648; doi:10.1371/journal.pone.0160971)
Supplement: S1 Appendix — (PDF) [file pone.0160971.s001.pdf]

Collaborating DPV centers

Augsburg Kinderklinik Zentralklinikum, Aachen - Uni-Kinderklinik RWTH, Ahlen St. Franziskus Kinderklinik, Aue Helios Kinderklink, Aurich Kinderklinik, Wien Uni-Kinderklinik, Weingarten Kinderarztpraxis, Berlin Lichtenberg - Kinderklinik, Berlin Virchow-Kinderklinik, Berlin Vivantes Hellersdorf Innere, Berlin Klinik St. Hedwig Innere, Berlin Schlosspark-Klinik Innere, Bad Aibling Internist. Praxis, Bremerhaven Kinderklinik, Bielefeld Kinderklinik Gilead, Bonn Uni-Kinderklinik, Braunfels-Wetzlar Innere, Hinrichsegen-Bruckmühl Diabetikerjugendhaus, Bottrop Kinderklinik, Bottrop Knappschaftskrankenhaus Innere, Celle Klinik für Kinder- und Jugendmedizin, Chemnitz Kinderklinik, Coesfeld Kinderklinik, Düsseldorf Uni-Kinderklinik, Darmstadt Kinderklinik Prinz. Margaret, Deggendorf Pädiatrie-Praxis, Deggendorf Medizinische Klinik II, Düren-Birkesdorf Kinderklinik, Delmenhorst Kinderklinik, Deggendorf Kinderklinik, Detmold Kinderklinik, Dortmund Kinderklinik, Dortmund-St. Josefshospital Innere, Dresden Uni-Kinderklinik, Datteln Vestische Kinderklinik, Essen Uni-Kinderklinik, Erlangen Uni-Kinderklinik, Erfurt Kinderklinik, Esslingen Klinik für Kinder und Jugendliche, Eutin St.-Elisabeth Innere, Eutin Kinderklinik, Frankfurt Uni-Kinderklinik, Offenbach/Main Kinderklinik, Freiburg Uni-Kinderklinik, Friedberg Innere Klinik, Friedrichshafen Kinderklinik, Fürth Kinderklinik, Fulda Kinderklinik, Gaissach Fachklinik der Deutschen Rentenversicherung Bayern Süd, Garmisch-Partenkirchen Kinderklinik, Gießen Uni-Kinderklinik, Göppingen Kinderklinik am Eichert, Gelsenkirchen Kinderklinik Marienhospital, Göttingen Uni-Kinderklinik, Görlitz Städtische Kinderklinik, Hannover Kinderklinik MHH, Hannover Kinderklinik auf der Bult, Hannover Henriettenstift - Innere, Halle Uni-Kinderklinik, Halle-Dölau Städtische Kinderklinik, Hachenburg Kinderpraxis, Hamm Kinderklinik, Bremen Zentralkrankenhaus Kinderklinik, Bremen - Kinderklinik Nord, Heilbronn Innere Klinik, Heidelberg Uni-Kinderklinik, Heidenheim Kinderklinik, Herford Klinikum Kinder &

Jugendliche, Bad Hersfeld Kinderklinik, Herzberg Kreiskrankenhaus Innere, Hermeskeil Kinderpraxis, Hagen Kinderklinik, Hamburg Altonaer Kinderklinik, Hamburg Kinderklinik Wilhelmstift, Hamburg-Nord Kinder-MVZ, Hildesheim Kinderklinik, Hildesheim Kinderarztpraxis, Lübeck Uni-Kinderklinik, Homburg Uni-Kinderklinik Saarland, Hanau Kinderklinik, Itzehoe Kinderklinik, Jena Uni-Kinderklinik, Köln Uni-Kinderklinik, Karlsruhe Städtische Kinderklinik, Kaiserslautern-Westpfalzklinikum Kinderklinik, Karlsburg Klinik für Diabetes & Stoffwechsel, Kempen Heilig Geist - Innere, Kiel Städtische Kinderklinik, Koblenz Kinderklinik Kemperhof, Koblenz Kemperhof 1. Med. Klinik, Krefeld Innere Klinik, Bad Kreuznach-St.Marienwörth-Innere, Kassel Klinikum Kinder- und Jugendmedizin, Leipzig Uni-Kinderklinik, Ludwigsburg Kinderklinik, Landshut Kinderklink, Lingen Kinderklinik St. Bonifatius, Lippstadt Evangelische Kinderklinik, Ludwigshafen Kinderklinik St.Anna-Stift, Lüdenscheid Märkische Kliniken - Kinder & Jugendmedizin, München von Haunersche Kinderklinik, München-Harlaching Kinderklinik, Mannheim Uni-Kinderklinik, Mannheim Uniklinik Innere Medizin, Marburg Uni-Kinderklinik, Mechernich Kinderklinik, Minden Kinderklinik, Moers Kinderklinik, Münster pädiat. Schwerpunktpraxis, Münster Uni-Kinderklinik, Mutterstadt Kinderarztpraxis, Nürnberg Zentrum f Neugeb./Kinder & Jugendl., Nagold Kreiskrankenhaus Innere, Neuwied Kinderklinik Elisabeth, Neunkirchen Marienhausklinik Kohlhof Kinderklinik, Nürnberg Cnopfsche Kinderklinik, Oberhausen Kinderklinik, Oberhausen Innere, Oldenburg Kinderklinik, Osnabrück Christliches Kinderhospital, Bad Oeynhausen Herz-und Diabeteszentrum NRW, Paderborn St. Vincenz Kinderklinik, Pforzheim Kinderklinik, Regensburg Kinderklinik St. Hedwig, Remscheid Kinderklinik, Mönchengladbach Kinderklinik Rheydt Elisabethkrankenhaus, Rendsburg Kinderklinik, Rosenheim Kinderklinik, Rastatt Kreiskrankenhaus Innere, Rastatt Gemeinschaftspraxis, Ravensburg Kinderklink St. Nikolaus, Rotenburg/Wümme Agaplesion

Diakonieklinikum Kinderabteilung, Stuttgart Olgahospital Kinderklinik, Saalfeld Thüringenklinik Kinderklinik, Saarlouis Kinderklinik, Saarbrücken Kinderklinik Winterberg, Schw. Gmünd Stauferklinik Kinderklinik, Suhl Kinderklinik, Siegen Kinderklinik, Singen - Hegauklinik Kinderklinik, Sinsheim Innere, Spaichingen Innere, Stade Kinderklinik, Sylt Rehaklinik, Trier Kinderklinik der Borromäerinnen, Ulm Uni-Kinderklinik, Vechta Kinderklinik, Viersen Kinderkrankenhaus St. Nikolaus, Weiden Kinderklinik, Wiesbaden Kinderklinik DKD, Wiesbaden Helios Horst-Schmidt-Kinderkliniken, Herdecke Kinderklinik, Waldshut-Tiengen Kinderpraxis Biberbau, Winnenden Rems-Murr Kinderklinik, Worms Kinderklinik, Wuppertal Kinderklinik, Kassel Städtische Kinderklinik, Magdeburg Uni-Kinderklinik, Schweinfurt Kinderklinik, Hildesheim GmbH - Innere, Idar Oberstein Innere, Saaldorf-Surheim Diabetespraxis, Neuss Lukaskrankenhaus Kinderklinik, München-Schwabing Kinderklinik, Passau Kinderklinik, Neuburg Kinderklinik, Augsburg IV. Med. Klinik, Scheidegg Reha-Kinderklinik Maximilian, Rostock Universität Innere Medizin, Bad Lauterberg Diabeteszentrum Innere, Recklinghausen Dialysezentrum Innere, Frankfurt Uni-Klinik Innere, Rosenheim Innere Medizin, Memmingen Kinderklinik, Herten St. Elisabeth Innere Medizin, Innsbruck Uni-Kinderklinik, Bad Kösen Kinder-Rehaklinik, Kaufbeuren Innere Medizin, Ludwigsburg Innere Medizin, Wetzlar Schwerpunkt-Praxis, Tübingen Uni-Kinderklinik, Heringsdorf Inselklinik, Marburg - UKGM Endokrinologie & Diabetes, Gelnhausen Kinderklinik, Asbach Kamillus-Klinik Innere, Merzig Kinderklinik, Lindenfels Luisenkrankenhaus Innere, Bad Reichenhall Kreisklinik Innere Med., Stolberg Kinderklinik, Münster St. Franziskus Kinderklinik, Passau Kinderarztpraxis, Berlin St. Josephskrankenhaus Innere, Bad Säckingen Hochrheinklinik Innere, Leverkusen Kinderklinik, Dornbirn Kinderklinik, Altötting Zentrum Inn-Salzach, Eberswalde Klinikum Barnim Werner Forßmann - Innere, Offenburg Kinderklinik, Kiel Universitäts-Kinderklinik, Kirchheim-Nürtingen Innere,

Herne Evan. Krankenhaus Innere, Duisburg Malteser Rhein-Ruhr St. Anna Innere, Mühlacker Enzkreiskliniken Innere, Neuwied Marienhaus Klinikum St. Elisabeth Innere, Rostock Uni-Kinderklinik, Wilhelmshaven St. Willehad Innere, Bocholt Kinderklinik, Oberhausen Kinderpraxis, Schwerin Kinderklinik, Fulda Innere Medizin, Rheine Mathiasspital Kinderklinik, Essen Elisabeth Kinderklinik, Mainz Uni-Kinderklinik, Traunstein diabetol. Schwerpunktpraxis, Villingen-Schwenningen Schwarzwald-Baar-Klinikum Innere, Herford Kinderarztpraxis, München-Gauting Kinderarztzentrum, Magdeburg Städtisches Klinikum Innere, Moers - St. Josefskrankenhaus Innere, Papenburg Marienkrankenhaus Kinderklinik, Wilhelmshaven Klinikum Kinderklinik, Bochum Universitätskinderklinik St. Josef, Köln Kinderklinik Amsterdamerstrasse, Heilbronn Kinderklinik, Chemnitz-Hartmannsdorf Innere Medizin - DIAKOMED-1, Graz Uni-Kinderklinik, München Diabetes-Zentrum Süd, Dortmund Knappschaftskrankenhaus Innere, Krefeld Kinderklinik, Rosenheim Schwerpunktpraxis, Reutlingen Klinikum Steinenberg Innere, Bad Waldsee Kinderarztpraxis, Aalen Kinderklinik, Duisburg Evang. und Johanniter Krhs Innere, Schwerin Innere Medizin, Wolgast Innere Medizin, Geislingen Klinik Helfenstein Innere, Kirchen DRK Krankenhaus Kinderklinik, Berlin DRK-Kliniken Pädiatrie, München 3. Orden Kinderklinik, Darmstadt Innere Medizin, Bautzen Oberlausitz KK, Hamburg Endokrinologikum, Freiburg Uni Innere, Rüsselsheim Kinderklinik, Trostberg Innere, Oy-Mittelberg Hochgebirgsklinik Kinder-Reha, Berchtesgaden CJD, St. Augustin Kinderklinik, Wernberg-Köblitz SPP, Tettnang Innere Medizin, Frankenthal Kinderarztpraxis, Dresden Neustadt Kinderklinik, Osterkappeln Innere, Berchtesgaden MVZ Innere Med, Haren Kinderarztpraxis, Bad Mergentheim - Gemeinschaftspraxis DM-dorf Althausen, Konstanz Kinderklinik, Waldshut Kinderpraxis, Gera Kinderklinik, Reutlingen Kinderarztpraxis, Heidelberg Uniklinik Innere, Arnsberg-Hüsten Karolinenhosp. Kinderabteilung, Schwäbisch Hall Diakonie

Kinderklinik, Oldenburg Schwerpunktpraxis, Hof Kinderklinik, Bremen - Mitte Innere, Kreischa-Zscheckwitz Klinik Bavaria, Linz Krankenhaus der Barmherzigen Schwestern Kinderklinik, Nauen Havellandklinik, Ulm Schwerpunktpraxis Bahnhofsplatz, Pirmasens Städtisches Krankenhaus Innere, Ingolstadt Klinikum Innere, Hameln Kinderklinik, Limburg Innere Medizin, Heide Kinderklinik, München Kinderarztpraxis diabet. SPP, Iserlohn Innere Medizin, Prenzlau Krankenhaus Innere, Dortmund Medizinische Kliniken Nord, Ulm Endokrinologikum, Linz Krankenhaus Barmherzige Schwestern Kardiologie Abt. Int. II, Bad Orb Spessart Klinik Reha, Weisswasser Kreiskrankenhaus, Frankfurt Diabeteszentrum Rhein-Main-Erwachsenendiabetologie (Bürgerhospital), Wittenberg Kinderklinik, Mödling Kinderklinik, St. Pölten Universitäts-Kinderklinik, Braunschweig Kinderarztpraxis, Berlin Endokrinologikum, Pfullendorf Innere Medizin, Kleve Innere Medizin, Bayreuth Innere Medizin, Böblingen Kinderklinik, Gießen Ev. Krankenhaus Mittelhessen, Mühldorf am Inn Kinderarztpraxis, Oschersleben MEDIGREIF Bördekrankenhaus, Plauen Vogtlandklinikum, Altötting-Burghausen Innere Medizin, Bad Salzungen Kinderklinik, Bad Mergentheim - Diabetesfachklinik, Erlangen Uni Innere Medizin, Reutlingen Kinderklinik, Villach Kinderklinik, Frankfurt Diabeteszentrum Rhein-Main-pädiat. Diabetologie (Clementine-Hospital), Lienz BKH Pädiatrie, Forchheim Diabeteszentrum SPP, Ulm Uni Innere Medizin, Salzburg Universitäts-Kinderklinik, Scheidegg Prinzregent Luitpold, Wien Preyersches Kinderspital, Offenbach/Main Innere Medizin, Kaiserslautern Kinderarztpraxis, München Schwerpunktpraxis, Wien 3. Med. Hietzing Innere, Leoben LKH Kinderklinik, Duisburg-Huckingen, Münster Herz Jesu Innere, Marktredwitz Innere Medizin, Wien Wilhelminenspital 5. Med. Abteilung, Wien SMZ Ost Donauspital, Wien Rudolfstiftung, Worms - Weierhof, Linz Landes-Kinderklinik, Wels Klinikum Pädiatrie, Duisburg Sana Kinderklinik, Villingen-Schwenningen Schwarzwald Baar Klinikum Kinderklinik, Amstetten Klinikum

Mostviertel Kinderklinik, Saarbrücken Kinderklinik Winterberg 2, Freiburg St. Josef Kinderklinik, Feldkirch Kinderklinik, Lappersdorf Kinderarztpraxis, Ludwigshafen diabetol. SPP, Oberhausen St.Clemens Hospitale Sterkrade, Dortmund-Hombruch Marienhospital, Dessau Kinderklinik, Luxembourg - Centre Hospitalier, Bad Orb Spessart Klinik, Neumarkt Innere, Bad Kreuznach-Viktoriastift, Lüdenscheid Hilfswerk Kinder & Jugendliche, Eisleben Lutherstadt Helios-Klinik, Lienz Diabetesschwerpunktpraxis für Kinder und Jugendliche, Olpe pädiatrische Gemeinschaftspraxis, Lilienthal Diabeteszentrum, Waren-Müritz Kinderklinik, Aidlingen Praxisgemeinschaft, Freiburg Kinder-MVZ, Heidelberg St. Josefskrankenhaus, Duisburg-St.Johannes Helios, Lienz BKH Kinderklinik, Essen Diabetes-Schwerpunktpraxis, Münster Clemens-Hospital Innere, Leer Kreiskrankenhaus - Kinderabt., Singen Kinderarztpraxis, Dinslaken Kinderklinik, Kempten Oberallgäu Kinderklinik, Freudenstadt Kinderklinik, Halberstadt Kinderklinik AMEOS, Halberstadt Innere Med. AMEOS Klinik, Bruchweiler Edelsteinklinik Kinder-Reha, Wismar Kinderklinik, Coburg Kinderklinik, Essen Kinderarztpraxis, Filderstadt Kinderklinik, Meissen Kinderklinik Elblandklinikum, Greifswald Uni-Kinderklinik, Ried Innkreis Barmherzige Schwestern, Wangen Oberschwabenklinik Innere Medizin, Traunstein Kinderklinik, Augsburg Josefinum Kinderklinik, Amberg Kinderklinik St. Marien, Zweibrücken Kinderarztpraxis, Neuruppin Kinderklinik, Bochum Universitäts St. Josef, Marburg Uni-Kinderklinik, Flensburg Diakonissen Kinderklinik, Frankfurt-Sachsenhausen Innere, Deggendorf Gemeinschaftspraxis, Oberndorf Gastroenterologische Praxis Schwerpunkt Diabetologie, Memmingen Internistische Praxis.
